# Heat Stress and Determinants of Kidney Health Among Agricultural Workers in the United States: An Integrative Review

**DOI:** 10.3390/ijerph22081268

**Published:** 2025-08-13

**Authors:** Justin J. Zhao, Erwin W. Leyva, Kamomilani A. Wong, Merle Kataoka-Yahiro, Leorey N. Saligan

**Affiliations:** 1 National Institute of Nursing Research, National Institutes of Health, Bethesda, MD 20892, USA; justin.zhao@nih.gov (J.J.Z.); erwin.leyva@norsu.edu.ph (E.W.L.); 2 National Kidney Foundation of Hawaii, Honolulu, HI 96814, USA; kwong@kidneyhi.org; 3 University of Hawaii at Manoa School of Nursing and Dental Hygiene, University of Hawaii, Honolulu, HI 96822, USA; merle@hawaii.edu

**Keywords:** heat stress, determinants of health, kidney health, agricultural workers

## Abstract

Agricultural workers in the United States (U.S.) are exposed to occupational heat stress, increasing their risk of adverse kidney outcomes. The aim of this integrative review was to explore the relationship between occupational heat stress and kidney health among U.S. agricultural workers. PubMed, EMBASE, Scopus, and Google Scholar were searched for original research articles on this relationship among U.S. agricultural workers. Studies were screened and reviewed by two independent reviewers in three phases: title and abstract screening, full text screening, and data extraction. The search yielded 278 articles; 14 were included in the final analysis. Heat stress was commonly measured using core body temperature changes, heat index, and wet-bulb globe temperature. Acute kidney injury (AKI) incidence following a single work shift was up to 43%. Occupational heat stress and piece-rate compensation increased the odds for developing AKI. The use of cooling bandanas and water mixed with electrolytes are promising interventions for mitigating the effect of heat stress on kidney health outcomes. The results confirm that occupational heat stress influences kidney health for U.S. agricultural workers. The mechanisms of this relationship have not been fully elucidated. More studies exploring heat protection interventions are needed.

## 1. Introduction

Agricultural workers in the United States (U.S.), who are hired labor that are engaged in agriculture as defined by the U.S. Department of Labor [1,2], consistently face work conditions that place them at elevated risk for adverse health outcomes. Among the most significant environmental hazards is occupational heat stress, which is defined by the U.S. Center for Disease Control and Prevention as a combination of metabolic and environmental heat, including from personal clothing and protective equipment, which results in increased storage of heat within the body [3]. Agricultural workers are particularly vulnerable to heat-related illnesses (HRIs) due to the labor-intensive nature of their work and prolonged exposure to high ambient temperatures, often while performing physically demanding tasks such as planting, weeding, hoeing/raking, irrigating, harvesting, and livestock care, frequently under direct sunlight or in greenhouses [4]. As global temperatures continue to rise, the frequency and severity of HRIs will increase as well, leading to heightened risks for adverse health events worldwide [5,6].

This workforce is largely composed of migrant and immigrant laborers, many of whom experience compounding socioeconomic stressors, including limited access to drinking water, health programs, and equitable working conditions [5,6]. They also tend to have lower levels of educational attainment, are more likely to be of Hispanic origin, and are less likely to hold U.S. citizenship compared to the general workforce [4]. These intersecting occupational and socioeconomic vulnerabilities contribute to heightened prevalence of health issues among agricultural workers, including occupational injuries, chronic conditions such as hypertension, and various infectious diseases [7]. Despite these medical risks, agricultural workers face substantial barriers to accessing healthcare, including lack of health insurance, high out-of-pocket costs, inflexible work schedules, language barriers, and limited availability of healthcare services [8]. Community and migrant health centers serve as the primary source of medical care for this population, yet many of these facilities are not fully equipped to address their health needs beyond basic primary care [7].

The relationship between occupational heat stress and negative health outcomes, particularly kidney health and disease, is becoming an increasingly recognized area of concern. This review will explore the relationships of occupational heat stress with acute kidney injury (AKI) and chronic kidney disease (CKD), as defined in the Kidney Disease: Improving Global Outcome (KDIGO) nomenclature guidelines [9]. The kidneys play a vital role in maintaining homeostasis, but high environmental temperatures and dehydration can decrease blood flow to the kidneys [10], which may reduce kidney function defined by a glomerular filtration rate (GFR) of less than 60 mL/min/1.73 m^2^ [9]. The KDIGO guidelines define kidney disease as structural or functional abnormalities of the kidneys that have health implications, where GFR and albuminuria categories are used to describe the severity of kidney disease [9]. Exposure to these environmental conditions can lead to AKI and, over longer periods, CKD without its accompanying traditional risk factors (e.g., hypertension, diabetes, family history of kidney disease, obesity, etc.) [11,12,13,14]. Most studies that explored the relationships of occupational heat stress and kidney health have mainly focused on tropical and low-to-middle-income countries, with few reporting on the issues within the U.S. [15]. As such, there is a need for studies that better elucidate the effects of occupational heat stress on kidney health outcomes of agricultural workers in the U.S.

Understanding the implications of occupational heat stress on kidney health is vital for developing effective medical interventions and informing policies that better address this public health concern. Recent systematic reviews and meta-analyses have been undertaken to address this gap on a global scale, giving insight into the link between high temperatures and kidney disease morbidity [11,16,17,18]. These studies underscore the need for further investigation into the associations and mechanisms behind this relationship. While examining global associations has its merits, future research can benefit from examining more localized geographies. Conducting national or even sub-national studies can generate new insights for each unique population. Furthermore, our study expands previous reviews conducted by including pathophysiologic and clinical processes, biological biomarkers, and interventions to address occupational heat stress among agricultural workers in the U.S.

This study primarily aims to provide an integrative review and synthesis of existing knowledge on the relationship between occupational heat stress and kidney health. Secondarily, the study aims to identify associated interventions to address occupational heat stress among agricultural workers in the U.S. We will build on the foundation set by previous reviews [19,20], which identified a variety of occupational risks such as inadequate protective clothing, lack of rest areas with shade, excessive workload, and more that place these agricultural workers at high risk for negative health outcomes and reduced kidney function. Our review will expand on these factors while placing additional emphasis on the social factors that further exacerbate health conditions [8,21,22]. Additionally, we will examine the efficacy of interventions such as promoting adequate water intake in protecting agricultural workers’ kidney health. In doing so, our literature review plans to identify patterns, highlight gaps in the literature, and uncover kidney-related health risks of U.S. agricultural worker populations exposed to occupational health stress. Insights from this review will inform the development of research questions to guide future investigations involving this population. Furthermore, we hope this review contributes to a call to action for more targeted research and improved occupational safety measures for this vulnerable population.

## 2. Materials and Methods

We conducted an integrative review of the literature to describe current evidence on the effects of occupational heat stress on kidney function among agricultural workers in the U.S., exploring biobehavioral and social factors, as well as interventions to reduce the effects of occupational heat stress. We followed the procedure suggested by Whittemore and Knafl, which included problem identification, literature search, data evaluation, data analysis, and data presentation [23].

### 2.1. Problem Identification

The following review questions were formulated: (1) What are the effects of occupational heat stress on the kidney function of agricultural workers in the U.S.?, (2) What are the physiologic, occupational, behavioral, and social factors that affect the associations of occupational heat stress and kidney function of these agricultural workers?, and (3) What interventions have been implemented and studied to protect agricultural workers from occupational heat stress?

### 2.2. Literature Search

**Figure 1:**
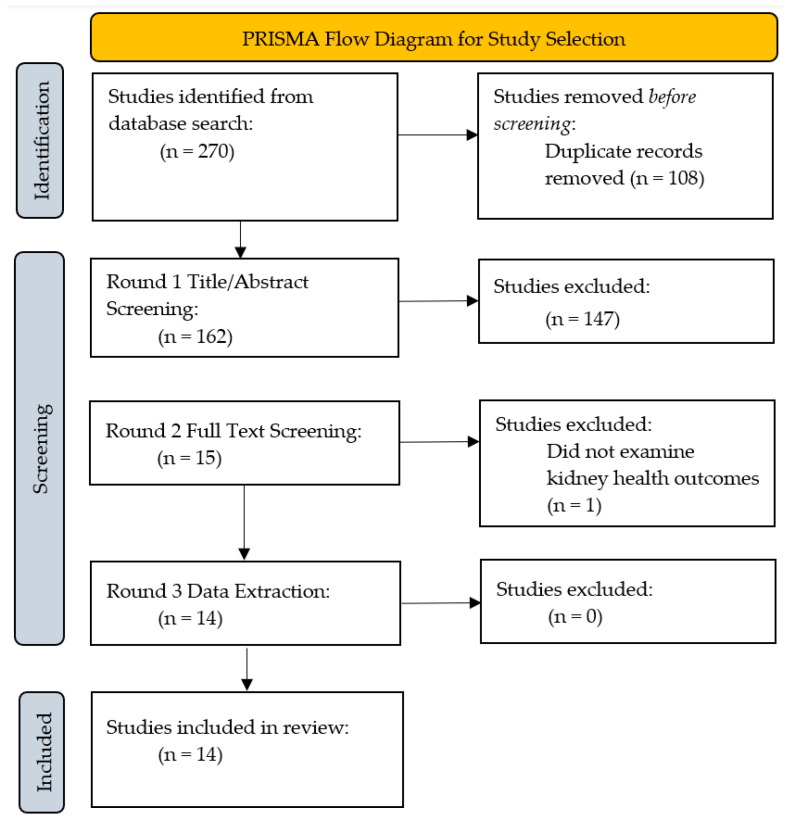
PRISMA flow diagram for study selection using Covidence.

An extensive literature search was conducted in electronic databases, including PubMed, EMBASE, Scopus, and Google Scholar, in consultation with a health librarian. We used the following search strategies: (farmers or farmworkers or “farm worker*” or “agricultural worker*”) and (heat or temperature or climate or weather or “global warming” or “Heat Stress Disorders” [Mesh]) and (kidney or renal). To determine the study’s eligibility, the following inclusion criteria were used: (1) research conducted with agricultural workers in the U.S.; (2) published in English; and (3) must address review aims. We excluded review articles, feature articles, editorials, and the grey literature. No limits pertaining to date of publication were used. Using various combinations of the key words, the search yielded a total of 278 articles. Removing duplicates reduced this number to 162. After applying the inclusion and exclusion criteria, 147 were removed due to irrelevance of the title and abstract to the topic of interest. Furthermore, we examined the full text of 15 articles and excluded 1 of the 15 for not focusing on kidney health outcomes. The literature search and review process are summarized in the PRISMA diagram (Figure 1). We used Covidence (Veritas Health Innovation Ltd., Melbourne, Australia) in the screening process (https://app.covidence.org, accessed on 8 August 2024).

### 2.3. Data Evaluation

Various reporting guidelines were used to describe the quality of the studies included in this review. These included CONSORT for randomized controlled trials [24], STROBE for observational studies [25], and CARE for case reports [26].

### 2.4. Data Analysis

We followed the elements of data analysis suggested by Whittemore and Knafl, which included noting patterns, seeing plausibility, clustering, counting, making contrasts and comparisons, discerning common and unusual patterns, subsuming particulars into general, noting relations between variability, finding intervening factors, and building a logical chain of evidence [23]. A literature review matrix was constructed containing information on study characteristics (i.e., author, year, purpose, design, state, sample, data analysis) and summarizing study findings based on the concepts of the PRECEDE-PROCEED model [27] (i.e., heat/weather measures, predisposers, enablers, reinforcers of heat protection, occupational risk factors, social factors, kidney health outcomes, and heat protection interventions) (Table 1).

## 3. Results

### 3.1. Characteristics of Studies

A total of 14 papers were included in the final analysis (Table 1) [28,29,30,31,32,33,34,35,36,37,38,39,40,41]. However, two of the articles came from one randomized clinical trial [36,38]. All authors and studies were based in the U.S., and studies were published in disciplines related to nursing, occupational and environmental health, and immigrant health. The publication date range of all studies was from 2016 to 2024. Most of the studies (n = 10 of 14) employed a cross-sectional design looking at one work shift (both pre- and post-shift). On the other hand, three studies used a longitudinal design where participants were followed over the course of several months to years. One of the publications was a case study that examined a single participant. Agricultural workers from only two unique states in the U.S. were recruited: Florida (n = 10) and California (n = 4). Apart from the one case study, sample sizes ranged from 30 to 471 participants. All studies enrolled both male and female participants to varying degrees (around 60–70% female), with only one employing a sample of nearly all male participants. The average ages of participants varied from 25 to 45 years old across the studies, with the majority being in their late 30s and early 40s. Many agricultural workers were either from the U.S., Mexico, or other Central American countries such as El Salvador or Guatemala. Participants worked either in ferneries, plant nurseries, field crops, or landscaping. For those studies that examined work duration, participants were full-time employees who worked at least 6 hours per day. None of the studies mentioned the size and ownership of the farms where study participants were employed or the income levels of the workers.

**Table 1:** Table listing the objective, study design, and key findings from each study in this review.

Author (Year)	Purpose	Design	State	Sample	Biomarkers of AKI/Dehydration	Key Findings
Moyce, et al. (2020) [28]	To test the associations between workload and heat with acute kidney dysfunction.	Cross-sectional measuring pre- and post-shift	California	471 agricultural workers	Pre- and post-shift serum creatinine	36% had elevations of core body temperature ≥ 1 °C. 14.9% had evidence of acute kidney injury (AKI) after a single day of work. Workload category was associated with increased odds of AKI (OR = 1.92, 95% CI = 1.05–3.51). Piece-rate work was associated with increased odds of AKI (OR = 3.02, 95% CI = 1.44–6.34).
Moyce, et al. (2017) [29]	To investigate the associations between heat strain, volume depletion, and kidney function	Cross-sectional measuring pre- and post-shift	California	283 agricultural workers	Pre- and post-shift serum creatinine	Thirty-five participants had an AKI incident after one shift. Heat strain was associated with increased odds of AKI (OR = 1.34, 95% CI = 1.04–1.74). Piece-rate work was associated with increased odds of AKI (OR = 4.24, 95% CI = 1.56–11.52).
Albu, et al. (2024) [30]	To evaluate the rate of retention and identify predictors associated with retention in a longitudinal study of agricultural workers	Longitudinal	Florida	119 agricultural workers	Pre- and post-shift serum creatinine; estimated glomerular filtration rate (eGFR)	One-third of workers reported HRI symptoms, and 15% demonstrated AKI. Appreciation for health tests was associated with greater participation at the final visit.
Mix, et al. (2018) [31]	To examine hydration status and kidney function in Florida agricultural workers	Longitudinal	Florida	192 agricultural workers	Pre- and post-shift serum creatinine, eGFR, and urine specific gravity	53% of workers were dehydrated pre-shift and 81% post-shift. 33% of workers had an AKI incident on at least one workday. Each 5 °F increase in heat index was associated with a 47% increased odds of AKI.
Houser, et al. (2021) [32]	To evaluate dehydration and markers of inflammation, muscle damage, and renal function in agricultural workers	Cross-sectional measuring pre- and post-shift	Florida	32 agricultural workers who self-identified as Hispanic	Pre- and post-shift serum creatinine; baseline urine specific gravity	Lower uromodulin and sodium levels in urine and elevated interleukin-6 and C-reactive protein (CRP) in serum were indicative of dehydration at baseline. Dehydration, body mass index (BMI), reduced uromodulin, and elevated serum interleukin-6, CRP, and lipopolysaccharide-binding protein were predictive of AKI on subsequent workdays.
Chicas, et al. (2024) [33]	To investigate the associations between renal function and AKI over time in U.S. agricultural workers	Longitudinal	Florida	115 agricultural workers	Pre- and post-shift serum creatinine, eGFR, and urine specific gravity	21% of participants who worked in ferneries (piece-rate compensation) experienced AKI in 2020, and that proportion rose to 43% by 2022. 11% of workers in nurseries (hourly compensation) experienced AKI at baseline, and the incidence remained stable over time.
Abasilim, et al. (2024) [34]	To investigate hydration status during typical workdays and identify risk factors associated with increased dehydration in migrant farmworkers in Florida	Longitudinal	Florida	111 agricultural workers	Pre-, mid-, and post-shift urine specific gravity	96.8% of post-shift urine specific gravity samples indicated potential dehydration. Dehydration significantly declined with age (β = − 0.078, 95% CI = 0.150−0.006) but increased with BMI (β = 0.016, 95% CI = 0.003–0.028) and wet-bulb globe temperature (WBGT) (β = 0.054, 95% CI = 0.044–0.064). No significant associations were found between dehydration and clothing worn, intake of employer-provided water, or crop units harvested during a shift.
Moyce, et al. (2020) [35]	To assess hydration choices during work shifts and investigate associations between rehydration with sugary beverages and AKI	Cross-sectional measuring pre- and post-shift	California	445 agricultural workers	Pre- and post-shift serum creatinine	The total volume agricultural workers drank was associated with increased odds of AKI (OR = 1.47, 95% CI = 1.09–1.99). No association was found between sugary drinks and AKI.
Chicas, et al. (2021) [36]	To examine the effectiveness of cooling devices at preventing agricultural workers from exceeding a core body temperature threshold of 38 °C and attenuating HRI symptoms	Cross-sectional measuring pre- and post-shift	Florida	84 agricultural workers	No biomarkers	The bandana group had lower odds of exceeding the threshold of 38 °C (OR = 0.7, 90% CI = 0.2–3.2). The vest group had higher odds of exceeding the threshold (OR = 1.8, 90% CI = 0.4–7.9). Using both cooling devices at once had a similar effect on core temperature as using the vest only (OR = 1.3, 90% CI = 0.3–5.6).
Moyce, et al. (2016) [37]	To investigate the cumulative incidence of AKI over one shift among California agricultural workers	Cross-sectional measuring pre- and post-shift	California	295 agricultural workers	Pre- and post-shift serum creatinine	11.8% of participants had an AKI incident after a work shift. Piece-rate compensation was associated with increased odds of AKI (OR = 4.52, 95% CI = 1.61–12.7).
Chicas, et al. (2021) [38]	To explore agricultural workers’ perceptions and experiences with the cooling devices from the pilot study	Cross-sectional post-shift	Florida	61 agricultural workers	No biomarkers	Participants from the bandana group reported that it was practical and did not interfere with their normal work routine. Participants in the vest group thought the vest was effective at cooling but had mixed reviews on its practicality while working.
Flocks, et al. (2018) [39]	To present a case of renal failure in a farmworker and illustrate how academic-community collaborations can be clinically beneficial	Case study	Florida	1 agricultural worker	Baseline creatinine	A referral from the Girasoles study involving chronic renal failure required eight independent surgeries and continued dialysis. The inclusion of health care providers in the study ensures accurate health information is conveyed to study participants. The involvement of a research team with active community partners is what made the extensive follow-up feasible.
Chicas, et al. (2023) [40]	To examine the impact of heat exposure on renal biomarkers and the metabolome among agricultural workers and non-agricultural workers	Cross-sectional measuring pre- and post-shift	Florida	63 agricultural workers and 27 non-agricultural workers	Pre- and post-shift serum creatinine, eGFR, and urine specific gravity	Median levels of pre-shift creatinine and osteopontin (*p* < 0.05) were higher for agricultural workers. Metabolic pathway enrichment analyses demonstrated 27 differing pathways between the two groups, including the tricarboxylic acid cycle, urea cycle, carbohydrate metabolism, and histidine metabolism.
Chicas, et al. (2022) [41]	To evaluate the impact of hydration interventions on post-workday hydration status and AKI incidence	Cross-sectional measuring pre- and post-shift	Florida	30 agricultural workers	Pre- and post-shift serum creatinine, eGFR, and urine specific gravity	No participants in the electrolyte group had an estimated glomerular filtration rate (eGFR) less than 90 mL/min/1.73 m^2^ or AKI incident compared to a normal workday (eGFR < 90 = 28%, AKI = 18%) or the water only group (eGFR < 90 = 15%, AKI = 23%).

All observational studies (n = 10) described at least 80% of the sections listed in the STROBE criteria [25], only missing some aspects of bias discussion (n = 4) [30,32,33,34] and generalizability to larger populations (n = 6) [28,29,32,33,34,40]. Randomized trials (n = 2) generally followed the CONSORT guidelines [24]; however, they did not report on any trial registrations (n = 2) [36,38,41] or participant flow (n = 1) [41]. The case report [39] followed CARE guidelines [26] and provided detailed information on interventions, follow-ups, and lessons learned.

### 3.2. Heat as a Climate Stressor

Heat as a climate stressor is associated with changes in the environment, such as increasing temperatures, that can negatively impact human health [42]. In these studies, it was evaluated as heat strain experienced by the individual or the general heat strain of the region where agricultural workers labored [28,29,30,31,32,33,34,35,36,38,39,40,41]. Individual heat strain was measured (35.7% of reviewed articles) using changes in core body temperature and heart rate [28,29,35,36,39]. Measurements of general heat stressors varied more with the use of the heat index. Heat index, which is commonly used in environmental health research as a combined measure of ambient moisture and temperature, was calculated during the participant’s work hours [31,32,33,34,36,38,41]. Other studies directly used ambient temperature and relative humidity [30,32,38]. Another occupational heat stress measure used that combines multiple aspects of the thermal environment similar to the heat index is the wet-bulb globe temperature (WBGT). Unlike the heat index, WBGT takes into account factors of ambient temperature, humidity, solar radiation, and wind speed [43]. However, only three studies utilized WBGT, whereas seven studies used heat index [34,35,40]. It was common for researchers to apply multiple measures to assess occupational heat stress, both regional and individual-specific, in the reviewed studies [32,33,34,35,36,38].

### 3.3. Health Status and Dehydration as Risk Factors

Across the studies reviewed, various predisposing factors to kidney disease vulnerability were examined. The majority of the studies (64.3%) considered traditional risk factors such as hypertension, diabetes, and a personal or family history of kidney disease [28,29,30,31,32,33,35,37,39]. Body mass index (BMI) was used frequently (64.3%) as a risk factor [30,32,34,35,36,37,39,40,41]. Interestingly, 14% of the reviewed studies assessed predisposing risk factors directly related to occupational heat stress, such as recent HRI symptoms or heat safety attitudes and behaviors [32,34]. Other health status-related risk factors were measured less consistently but mainly focused on hydration. Volume depletion, calculated by the differences between pre- and post-shift body weight, was the most common method (21.4%) of quantifying acute dehydration status of workers [28,29,35]. In addition, some studies (35.7%) assessed hydration behaviors of agricultural workers, such as the type and volume of beverages consumed both at home and at work [31,33,35,40,41]. Only Chicas et al. (2024) accounted for reinforcers of heat protection when asking workers about previous training in HRI prevention [33].

### 3.4. Occupational Risk Factors

Agricultural workers face a variety of occupational risk factors that can exacerbate the effects of occupational heat stress on kidney function. The most common factors examined were daily working conditions at the work location (fernery, nursery, or field crops at 28.6%) [30,31,38,41], duration of shifts (35.7%) [31,34,38,40,41], and whether workers had access to clean water and shade (21.4%) [31,33,34]. Many studies (35.7%) also assessed the primary task performed by agricultural workers during their shift [28,29,34,35,37]. Apart from occupational risk factors related directly to the agricultural workers’ day-to-day conditions, many researchers (71.4%) also accounted for how many years the participants had worked in the agricultural industry [28,29,30,31,33,35,36,37,40,41]. Piece-rate work, which means workers are paid by how much they produce rather than an hourly or fixed wage, was consistently (28.6%) associated with increased odds of developing AKI as opposed to hourly or fixed wages [29,33,35,37]. One study also found that 43% of participants working in ferneries developed AKI in 2022 as opposed to around 11% of participants who worked in nurseries [33].

### 3.5. Social Factors

Sociodemographic factors such as age, sex, nationality, language, and level of education were often recorded across the studies (78.6%) [28,29,30,31,32,33,34,35,38,40,41]. Income was not assessed in the reviewed studies; however, sex was found to be associated with differences in developing AKI. Moyce et al. (2017) found that in workers paid by the piece, females had 102.81 adjusted odds of AKI [29]. In addition to sex, three studies reported associations between these sociodemographic factors and retention in longitudinal studies and kidney health outcomes. Older individuals and those of Mexican nationality were more likely to remain interested in study participation [30]. Both Abasilim et al. (2024) and Mix et al. (2018) found that urine specific gravity levels significantly declined with age [31,34].

### 3.6. Pathophysiologic Processes

The pathophysiology of kidney dysfunction in agricultural workers was explored in a few studies. Heat strain, measured via changes in dehydration and core body temperature, was found in 57.1% of the reviewed articles associated with physiological markers of kidney dysfunction such as elevated creatinine and urine specific gravity [28,29,30,31,32,33,34,37]. Houser et al. (2021) attempted to further explore the relationships between potential serum biomarkers and AKI in participants working under high-temperature conditions. They demonstrated that agricultural workers who were dehydrated and had reduced urine uromodulin and elevated serum interleukin-6, C-reactive protein, and lipopolysaccharide-binding protein at baseline were predictive of experiencing AKI on subsequent workdays [32]. Similarly, Chicas et al. (2023) investigated the impact of heat exposure on renal biomarkers and metabolic pathways, finding that pre-shift creatinine and osteopontin levels were higher in agricultural workers with heat exposure compared to workers without. Additionally, Chicas et al. (2023) identified that metabolic pathways in the tricarboxylic acid (TCA) cycle and urea cycle, which are involved in normal kidney function, were perturbed in agricultural workers and associated with WBGT [40].

### 3.7. Kidney Health Outcomes

The kidney health outcome examined in all the reviewed studies was focused on AKI. It was also found that increased workload and heat strain were associated with higher odds of developing AKI, with a 47% increase for each 5° increase in heat index [28,29,31]. Interestingly, Moyce et al. (2020) found that higher amounts of fluid intake were associated with increased odds of AKI [35]. Urine specific gravity measures also indicated that between 81% and 96.8% of workers were potentially dehydrated post work shifts, and that increases in urine specific gravity levels were associated with WBGT and shift duration [31,34].

### 3.8. Heat Protection Interventions for Agricultural Workers

A few studies (21.4%) focused specifically on the effects of heat protection interventions to address occupational heat stress. In a pilot study by Chicas et al. (2023), they found that the use of a cooling bandana significantly lowered the odds of exceeding a core temperature of 38 °C, while the use of a cooling vest demonstrated the opposite effect [36]. They further evaluated the feasibility of incorporating these cooling devices into daily working use. Many participants agreed that the bandana was both practical at cooling and did not interfere with work. Those that used the vest reported that it was effective at cooling them but was not entirely practical for use while working due to the weight [38]. In a separate pilot study, Chicas et al. (2022) were the first to explore hydration interventions in U.S. agricultural workers. They found that drinking 5 L of water mixed with electrolytes can be effective in preventing decreased estimated glomerular filtration rates and AKI incidence compared to plain water [41]. Flocks et al. (2018) also identified that improving access to general health screenings and personal health care through referrals for specialty follow-ups are important factors in incentivizing agricultural workers to participate in future research on heat protection interventions [39].

## 4. Discussion

**Figure 2:**
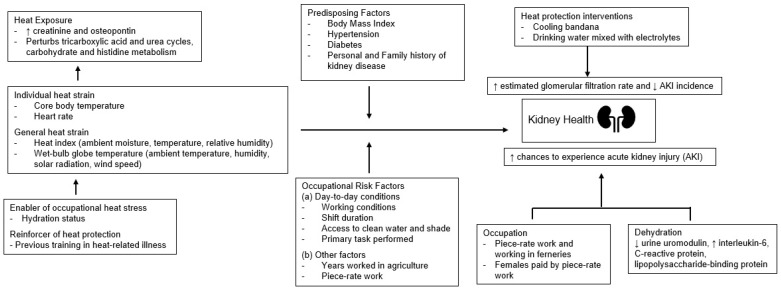
Visual representation of the main findings reporting on the relationship of occupational heat stress and kidney health outcomes. Arrows (↑, ↓, →) indicate the direction of relationships between variables or the direction of effects of specific factors or interventions.

This integrative review consolidated information from 14 studies published between 2016 and 2024 that examined relationships between occupational heat stress and determinants of kidney health among U.S. agricultural workers. There is strong evidence that workers experience excessive occupational heat stress, which contributes to AKI [28,29,31,34], and is exacerbated by both traditional [32,34] and occupational risk factors [28,29,33,37,40]. Preliminary evidence indicates that inflammatory biomarkers and perturbations to metabolic pathways in the TCA and urea cycles may influence kidney health in this context [32,40]. Our findings also show that heat protection interventions ranging from clothing to hydration strategies have demonstrated promising results and highlight the importance of continuing research in this domain [36,38,41]. A visual summary of the results can be found in Figure 2.

A recurring finding across studies was a high incidence rate of AKI among U.S. agricultural workers after just one full (≥6 h) shift [28,29,30,31,37,41]. Definitions of AKI were also relatively consistent, with many relying on ≥3 mg/dL or ≥1.5 times baseline increases in serum creatinine post-shift [28,29,30,31,32,33,35,37], allowing for the comparison of incidence rates across studies. Increased risk of developing AKI was observed in workers who received piece-rate compensation [28,29,37], which meant they were paid by how much they produced rather than a fixed wage. As a result, some workers may feel the need to overexert themselves and skip breaks so as to not reduce their income [44], potentially working past the onset of initial HRI symptoms. One study also found that females paid by how much they produce had significantly higher odds of AKI compared to males paid by the same method [29]. Although this was only reported by one study, it warrants further investigation to better determine how sex-based differences can affect kidney health outcomes in an agricultural worker population. Other variables, such as elevated urine specific gravity to measure dehydration, were associated with age [34]. This result is consistent with the previous literature reporting that urine specific gravity measurements are affected by sociodemographic factors and that increases in age are associated with decreases in urine specific gravity [45]. This association could potentially explain the difference in urine specific gravity levels rather than intentional hydration choices in older agricultural workers enrolled in these studies.

However, it is clear that dehydration, as indicated by elevated urine specific gravity levels, is associated with higher WBGT [34]. Increasing environmental temperatures, coupled with the fact that agricultural workers may not drink enough water to stay adequately hydrated [46], may exacerbate water loss and dehydration. Interestingly though, one study found that increased fluid intake was positively associated with AKI [35]. This result is not unique, as a few previous studies have demonstrated a similar finding that increased water consumption was associated with elevated odds of CKD or renal insufficiency [47,48]. Researchers have hypothesized that this could potentially be due to the ingestion of contaminated water or a pre-existing kidney issue in concentrating urine [35,47], although further investigation is needed to identify the causes.

Urine specific gravity is a common method to assess dehydration, and the reviewed studies all used the American College of Sports Medicine recommendation of >1.020 cut-off as a marker [49]. There may be inconsistencies with the use of urine specific gravity as a marker of dehydration if measured during a person’s rehydration period [50]; however, it still remains as a useful marker due to its practicality and quantifiability [49]. Serum creatinine was consistently used as a biomarker in all reviewed studies that assessed AKI. In these instances, AKI was defined as an increase in post-shift creatinine level by at least 0.3 mg/dL or 1.5 times the pre-shift level, as supported by the KDIGO guidelines [51]. However, the use of serum creatinine is limited by delayed changes in creatinine levels following kidney injury. Steady-state levels required for accurate creatinine quantification may not be achieved within a clinically relevant timeframe, and its lack of specificity for kidney function constraints its clinical utility [52]. As such, there have been recommendations to use creatinine in conjunction with other biomarkers, including cystatin C and urine neutrophil gelatinase–associated lipocalin (NGAL), along with clinical assessments of urine output, to more properly diagnose AKI [53]. Future studies examining kidney health using AKI incidence should consider these recommendations to improve the accuracy of diagnoses.

Despite the need for preventive strategies in addressing occupational heat stress for this population, intervention studies are limited. Chicas et al. (2021) was the only group to investigate the impact of personal cooling devices and hydration on both heat exposure and downstream kidney outcomes. The use of bandanas showed physiological benefits in lowering core temperature with little impact on work productivity and could be a potential lead for the development of clothing interventions [36,38]. A separate pilot study from the same group (2022) found that the mixing of electrolytes with water protected agricultural workers from post-shift AKI as well [41]. While these interventions show promise, their small and unbalanced sample sizes and short treatment periods limit the generalizability and longitudinal applicability of their findings. Additionally, workers may find it difficult to negotiate these changes in working conditions with employers [5]. As exploratory studies, these interventions were not evaluated in the context of employer implementation, highlighting the need for further research to fill the gap between experimental interventions and occupational policy changes.

Furthermore, the implementation of heat protection interventions should take into account the health knowledge of its users. Both Albu et al. (2024) and Flocks et al. (2018) identified that the retention of agricultural workers in longitudinal studies was associated with access to health screenings and personal health knowledge [30,39]. Although many participants may not receive formal HRI-related training, many workers are aware that excessive heat exposure causes health issues in their occupation [46,54]. Despite this, some workers may not fully utilize water resources, shade, and breaks that reduce occupational heat stress [55]. Improving personal knowledge on heat protection represents another avenue of interventions that can protect against occupational heat stress and adverse kidney outcomes. In fact, a previous study has demonstrated that participatory education can improve agricultural worker knowledge about occupational heat stress [56]. Therefore, future research on heat protection interventions for agricultural workers in the U.S. may benefit from combining personal and employer-level interventions. Nerbass et al. (2017) effectively summarized preventive strategies against occupational heat stress, emphasizing the need to integrate environmental monitoring such as early warning systems and surveillance to guide work-rest cycles with access to essential facilities (e.g., toilets) and resources (e.g., appropriate clothing, personal cooling methods), and stressing the importance of fair compensation, as income is a powerful motivator that may lead workers to overlook signs of heat strain [57].

Some methodological gaps emerged from this review. First, despite CKD and adverse kidney outcomes related to occupational heat stress and dehydration being explored in various Central American countries [58,59], there are no CKD studies that investigated this relationship before 2016 in the U.S. This could be explained by the lack of diagnoses specifically related to these outcomes inside the U.S. until 2015, when cases began appearing [13,60]. There was a geographic concentration of studies based in California and Florida, with no other states being examined. Although California and Florida do employ a high number of agricultural workers, these studies did not account for other states that may have similar levels, such as Oregon, Idaho, and Washington [61]. This limits the generalizability of these conclusions in other regions where policy and climate conditions differ. Second, although WBGT was established as an international standard measure for safe heat exposure, its use in day-to-day monitoring is not practical because WBGT requires specific and expensive equipment [57]. Another recommended measure is the Predicted Heat Strain (PHS) index, which can predict both sweat rate and internal core temperature resulting from heat stress [62]. Although both WBGT and PHS are not valid for the assessment of rapidly changing environments and short exposure durations [63]. A previous review suggested that future occupational heat stress assessments should take individual vulnerabilities into consideration and allow for a wide range of environmental and personal adjustments [64]. For example, these adjustments can include the provision of wearable microclimate cooling devices or policies that promote frequent and longer rest periods to reduce work intensity [64]. Third, there was a lack of reporting on the size and ownership of the farms where these studies took place, as well as the income levels of their workers. Furthermore, although many studies addressed AKI pre- and post-shifts, there has been little research on long-term kidney disease outcomes. Longitudinal outcomes are particularly prevalent, as studies in recent years have shown the development of chronic kidney disease of unknown etiology (CKDu) in agricultural workers across the globe [65,66]. Since AKI is a risk factor for CKD and end-stage renal disease [67], coupled with the associations between occupational heat stress and AKI shown here, it is possible that repeated occupational heat exposure in this population contributes to the development of CKDu. These repeated episodes of subclinical kidney injury can, over time, lead to permanent kidney injury, as captured in the heat-stress hypothesis summarized during the First International Research Workshop on the Mesoamerican Nephropathy [68]. Additionally, only one study incorporated pesticide or agricultural chemical exposure data despite previous studies that have suggested a link between pesticide exposure and CKDu [69]. Although the First International Research Workshop on the Mesoamerican Nephropathy identified heat stress as a primary factor and pesticide exposure as an alternative consideration [68], evidence has since linked pesticide exposure to both acute and chronic kidney outcomes [70,71,72].

Taken together, there are several avenues for future research that can address these gaps in the literature. Future research agendas focused on intervention studies should utilize larger sample sizes, an equal representation of male and female participants, and a larger distribution of ages to increase the generalizability of findings. At the same time, a longitudinal intervention trial or a longitudinal observational study conducted over an entire summer or harvesting season, rather than a single work shift, should be considered to better assess the impact of interventions and the influence of contributing factors on the incidence of AKI over time and the potential development of CKD. This approach will yield valuable insights into the relationship of repeated episodes of AKI and the subsequent development of CKD. Incorporating more detailed data on agricultural chemical exposures, employer characteristics, and income levels will enable future studies to more precisely delineate the impact of occupational and socioeconomic factors on the etiology of AKI and CKD.

There are also strengths and limitations in the use of an integrative review. The broad scope of the integrative review means that diverse methodologies, including experimental and non-experimental research, can be included. However, such a broad range of studies also means that a variety of variables can be captured, making data extraction difficult. There needs to be a balance between including results that can be compared across studies and providing specific results; ultimately, this can limit the granularity of information retrieved from individual studies. Although the studies included as part of this integrative review were of good quality according to reporting guidelines, there were some aspects of those guidelines that were underreported. Specifically, methods of accounting for bias were often excluded from studies [30,32,33,34] or only mentioned briefly [28,29,31,40]. Although a common technique for population research, the use of convenience samples could have introduced motivation bias into studies and may not necessarily capture the entire array of agricultural workers [73]. Several studies also commented that the conclusions drawn from the results may not be applicable to the entire agricultural worker population due to the fact that the employers who allowed workers to participate in a study on kidney health may have better working conditions compared to other employers who did not [28,29,35,37]. Despite the lack of reporting on specific patient flow for the randomized trial study [41], this was conducted using a small sample size within two work shifts, which meant there could have been no participant withdrawal, and the authors did not feel the need to report. Furthermore, there was no reporting of trial registrations due to their nature as pilot studies [36,38,41], and the guidelines of some journals that do not require the registration of these studies [74]. However, future randomized controlled trials that build on this research should consider these aspects in their design and reporting.

## 5. Conclusions

There has been increasing evidence over the past decade that occupational heat stress increases the risk of adverse kidney outcomes for U.S. agricultural workers. The risk of developing AKI can be further exacerbated by occupational risk factors such as piece-rate work and work type or sociodemographic factors. Although the mechanisms behind these changes in kidney health are not well characterized, they can be linked to changes in inflammatory and metabolic pathways important to kidney function. To date, few studies have attempted to examine hydration or clothing interventions in this population. However, these pilot studies demonstrate potential in reducing occupational heat stress and improving kidney health outcomes for U.S. agricultural workers. Further research is required to delineate the pathophysiology and effectiveness of heat protection interventions behind occupational heat stress and kidney health determinants. In addition to this research, there is a clear need to continue advocating for improved safety and policy standards for U.S. agricultural workers to tackle the risk of adverse kidney outcomes.

## Abbreviations

The following abbreviations are used in this manuscript:

AKI	Acute kidney injury
HRI	Heat-related illness
CKD	Chronic kidney disease
KDIGO	Kidney Disease: Improving Global Outcome
GFR	Glomerular filtration rate
CRP	C-reactive protein
BMI	Body mass index
WBGT	Wet-bulb globe temperature
TCA	Tricarboxylic acid
eGFR	Estimated glomerular filtration rate
PHS	Predicted heat strain
CKDu	Chronic kidney disease of unknown etiology
